# Molecular Effectors of Photodynamic Therapy-Mediated Resistance to Cancer Cells

**DOI:** 10.3390/ijms222413182

**Published:** 2021-12-07

**Authors:** Eric Chekwube Aniogo, Blassan P. George, Heidi Abrahamse

**Affiliations:** Laser Research Centre, Faculty of Health Sciences, University of Johannesburg, P.O. Box 17011, Doornfontein 2028, South Africa; ericaniogo@gmail.com (E.C.A.); blassang@uj.ac.za (B.P.G.)

**Keywords:** cancer cells, drug resistance, photodynamic therapy, autophagy

## Abstract

Photodynamic therapy (PDT) is currently enjoying considerable attention as the subject of experimental research to treat resistant cancers. The preferential accumulation of a non-toxic photosensitizer (PS) in different cellular organelles that causes oxidative damage by combining light and molecular oxygen leads to selective cell killing. However, one major setback, common among other treatment approaches, is tumor relapse and the development of resistance causing treatment failure. PDT-mediated resistance could result from increased drug efflux and decreased localization of PS, reduced light exposure, increased DNA damage repair, and altered expression of survival genes. This review highlights the essential insights of PDT reports in which PDT resistance was observed and which identified some of the molecular effectors that facilitate the development of PDT resistance. We also discuss different perceptions of PDT and how its current limitations can be overturned to design improved cancer resistant treatments.

## 1. Introduction

The use of sunlight exposure to treat various diseases such as skin diseases, diabetic ulcers, and epithelioma has been traced back to 3000 B.C. [1,2]. The modern use of light in a medical treatment known as phototherapy is producing a successful result in cancer treatment. Light within the infrared region, electromagnetic and artificial light sources are often used in phototherapy. Photodynamic therapy (PDT) is a non-invasive therapy that involves using light and a photoactive compound in the presence of molecular oxygen to produce a reactive oxygen species that damages cancer cells. This therapy was discovered by Prof. Hermann Von Tappeiner and groups over a century ago [3]. Since then, photodynamic reactions of light, photosensitive dye (called photosensitizer), and oxygen have gained popularity among other types of therapies. PDT is a promising therapeutic approach for various cancers and non-cancerous diseases such as age-related macular degeneration, atherosclerosis, and bacterial infections. 

In most cases, PDT treatment minimizes the need for surgery. It promotes good healing efficacy by conserving the integrity and function of organs with a relative risk of localized side effects [4]. Evidence has shown that PDT can be repeated with better cosmetic outcomes, minimal functional disturbances, good tolerance, fertility preservation, and low systemic toxicity. These are some of the advantages of PDT over the classic treatment strategies such as surgery, chemotherapy, and radiotherapy [5]. However, some drawbacks remain with the development of treatment resistance and tumor regrowth. The resistance of cancer cells to apoptosis is a fundamental aspect of cancer development and a major threat to most treatment strategies [6].

Resistance development of cells treated with PDT may arise due to poor photosensitizer localization, inadequate illumination, and reduced generation of reactive oxygen species (ROS). Additionally, tumor progression after PDT could result from increased drug efflux transporters expression, DNA repair mechanisms, decreased drug activation, and altered expression of tumor survival genes [7,8]. PDT aside, its resistance development has, over the years, proven to be an effective form of cancer therapy and a subject of numerous studies in bacteria inactivation. However, it is still underutilized and always meets a tough clinical trial approval for resistance to cancer treatment. This review provides an overview of PDT modality and molecular effectors that influence its treatment resistance. Most recent efforts made in the limitations of PDT and its use in resistant treatment and future direction will be given. Hence, we will analyze the molecular effector that aids PDT resistance and give insights into how these limitations can be overturned for a better PDT design for resistant cancer treatments.

## 2. Biochemical Effects of PDT and Reactive Oxygen Species (ROS) Production

PDT treatment modality requires the combined action of PS, light in red or near-infrared regions, and molecular oxygen to elicit cancer cell death. The cell death response of PDT depends on PS localization, light dose, and availability of molecular oxygen [9]. PS predominantly localizes in organelles such as plasma membrane, lysosomes, mitochondria, Golgi apparatus, or endoplasmic reticulum [10,11]. Under appropriate conditions and with the required light dose, PS placed at the target site is activated by absorbing photons of light energy and transferring it to in situ molecular oxygen to form a cytotoxic reactive oxygen species (ROS) [5]. The photon absorption by the PS will convert the ground state (S_0_) PS to an excited unstable state (S_1_) which will undergo intersystem crossing to a triplet (T_1_) state. The T_1_ state will then react with molecular oxygen and decay to S_0_ upon ROS formation. Or rather, the photon transfers from S_0_ to other reactive precursor molecules to form a radical species molecule that is cytotoxic [12]. In either case, the radicals produced have a strong reactivity with lipids, nucleic acids, proteins, and other biochemical substrates that lead to the activation of distinct tumoricidal mechanisms. Singlet oxygen is the principal product of type II photochemical reaction that causes PDT cell killing [13]. 

The photodamage caused by ROS can lead to apoptosis, necrosis, or autophagy, the three main mechanisms of cell death [14]. The phase of death activated after PDT treatment is determined by cell type, PDT dosimetry (e.g., light intensity), and PS type. The PS within the mitochondria stimulates apoptosis, whereas in the plasma membrane, it can initiate necrosis upon irradiation [15]. Overall, cell death initiation by PDT shifts from apoptotic to necrotic as the cell damage increases [15]. 

Apoptosis is a highly regulated cell death mechanism. It involves the initiation of numerous pathways following the damages of several organelles [16]. Mitochondria play a key role in apoptosis regulation and any PS localized on the organelle triggered the process. Leakage of the cytochrome c from the mitochondria into the cytosol can activate caspase proteins to induce signal transduction that leads to apoptosis [16,17]. Cell death by necrosis is extensive damage of cell components at PS site of action that results in leakage of intracellular material that can cause inflammation. This process usually occurs when PS localizes in the plasma membrane and PDT with higher doses of light and PS [18]. Beclin-1 is an essential tumor suppressor protein associated with autophagy. The autophagic process is multifaceted in action; the production of Beclin-1 targets tumor growth suppression. However, as the tumor becomes more advanced and progressing, an alternative process of autophagy is triggered to support the cells in the central, low nutrient part of the tumor to obtain the energy they need to stay alive [19]. In the later process, the photodamaged cells recycle their damaged organelles and cytoplasmic components to become resistant to PDT [13]. Depending on PDT dosage, autophagy can either be activated to recycle the damaged organelle to cause cell survival (low dose PDT) or cause complete cell organelle damage (high dose PDT) to induce cell death. However, mild PDT caused by insufficient localization of PS, efflux of PS out of the cell and low light prompts protective capacity of autophagy to repair the damage and thus lead to PDT resistance [20,21]. Mechanisms of autophagic response that triggered PDT resistance will be discussed below. 

## 3. Autophagy-Mediated PDT Resistance in Tumor Cells 

PDT effect can induce apoptotic or non-apoptotic cell death depending on the PS and light dose used [22]. PS localization in the lysosome causes photo-degradation toxicity of the lysosome upon irradiation and subsequently leads to cell death. PDT produces enough ROS that can initiate apoptotic cell death. In cases where ROS production is not sufficient, autophagy is triggered in response to cell survival and proliferation. Researchers have found that upregulation of *Bcl-2* protein protects the cells from the PDT mediated phototoxicity [23]. PDT-induced autophagy sometimes occurs before apoptosis through cytoplasmic content sequestration regulated by type I and III phosphoinositol-3-kinase proteins [24,25,26]. After PDT treatment, resistant murine cells were found to overexpress Bcl-2 anti-apoptotic protein and undergo non-apoptotic cell death (type II autophagy) [27]. However, other reports stated that *Bcl-2* protein tends to bind to the Beclin-1 to inhibit PDT resistance autophagic response [28]. Strategies such as gene silencing, especially *ATG5* gene by a pharmacological agent, have been reported as an effective treatment for resistant tumor cells [20]. 

**Figure 1:**
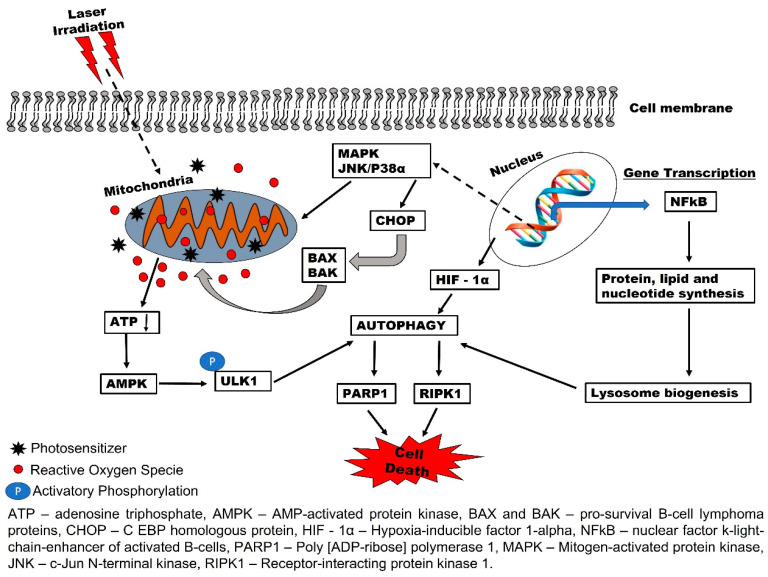
Autophagy-mediated response to photodynamic therapy (PDT). Photosensitizer activation in the mitochondria enhances the formation of ROS and decreases ATP production. Energy sensing AMPK activates the ULK1 to initiate autophagy. PDT can also trigger autophagy machinery *NFkB* for protein, lipid, and nucleotide synthesis to initiate lysosome biogenesis and autophagy. Mitochondria photo-oxidation can transcriptionally regulate autophagy through *MAPK*, *CHOP*, and *HIF—1α*.

The PDT efficacy was increased in *ATG5* knock-out HeLa and MCF-7 cells [29]. They also reported that photofrin-based PDT was very effective when the autophagic genes were inhibited. This suggests the role of autophagy in the therapeutic survival response of cells treated with PDT [29]. Many other inhibitors, such as 3-methyladenine, bafilomycin A1, obatoclax, clarithromycin, chloroquine, and hydrochloroquine, etc., have the same ability to block the cytoprotective effect of autophagy [30]. In response to PDT photodamage, some cellular contents are degraded and recycled to initiate the pro-survival mechanism of autophagy (Figure 1) [31]. In this context, activated photosensitizer in the mitochondria leads to the formation of reactive oxygen species (ROS) and a decrease in the adenosine triphosphate (ATP) production. The AMP-activated protein kinase (*AMPK*) senses the decrease in ATP and activates autophagy-initiating kinase 1 for reprogramming the metabolism of autophagy. Other reports have stated that cytoplasmic photodamage of PDT enhances the activation of NFkB and autophagy through *HIF-1α/VIMP1*-mediated [32] and *MAPK*1/3 [33] regulatory pathways (Figure 1). Moreover, factors such as nutrient depletion and oxidative stress injury from PDT ROS generation could contribute to the autophagic response. 

Mitophagy is another mechanism that tumor cells usually use as a rescue cellular homeostasis to compensate for mitochondrial photodamage of PDT. Mitophagy is a selective type of autophagy that functions as a negative regulatory feedback mechanism that reduces the mitochondrial-derived ROS production and prevents the release of proapoptotic proteins to abort cell death [34,35,36]. Nonetheless, excessive ROS generation of PDT causes the recruitment of ubiquitin ligase *PRKN/*parkin to initiate the degradation of the mitochondria through a process called mitophagy [34,37].

Additionally, the photo-stress of autophagy has been reported to cause excitation of the LC3-II proteins which oxidize the ER subdomains to cause *ATF4* or *CHOP* protein release. These proteins regulate the expression of autophagic proteins such as *ATG5*, *ATG12*, and Beclin 1 [38,39,40]. PDT damage to mitochondria, lysosomes, or endoplasmic reticulum organelles has shown to upregulate Bcl-2, and Bcl-xL anti-apoptotic and autophagy-related (*ATG5* and Beclin-1) proteins to induce PDT-resistance [41,42]. 

More evidence of autophagy and PDT interaction continue to accumulate despite the controversial findings of autophagy activation by excess ROS generation. It is now established that PDT ROS production triggers cellular damage to cytoplasmic organelles. This consensus may serve to delineate the underlying mechanistic relationship of autophagic death and survival following PDT [43]. 

Nevertheless, one major question remains: how PDT protocols with different PSs and cell lines differ in the therapeutic modulation of autophagic response. To answer this question and avoid the controversial analysis of the real role of autophagy (i.e., cytoprotective versus death routine), one must take into consideration the complexity of autophagy and numerous steps involved; hence, more research is needed in that aspect to arrive at a rational conclusion. 

## 4. Involvement of Pro-Survival Apoptotic Proteins in Photodynamic Therapy Resistance 

The *Bcl-2* family proteins consist of anti-apoptotic *(Bcl-XL, Bcl-W, A1*, and *Mcll*) and pro-apoptotic (*Bax* and *BH3*-only) proteins [16]. PDT initiates apoptosis by releasing mitochondrial cytochrome c into the cytosol following the activation of the apoptosome and pro-caspase 3. These proteins activate the Smac/DIABLO and Omi/HtrA2 by eliminating the inhibitory effects of caspase-3 and 9, which will cause the release of apoptosis inducing factor and endonuclease G into the cytosol to translocate into the nucleus and damage the DNA. This intrinsic pathway activation and overall apoptotic process of PDT integrates with the Bcl-2 family proteins and external cell death ligands such as *FasL, TNF-*α, and *TRAIL* [15]. 

PDT induces its photodamage on cells by varying measures such as upregulation of Bcl-2 and downregulation of *Bax*, which has been observed in resistant HT29 human colon adenocarcinoma cells [44]. On the contrarily, suppression of the *Bcl-2* mRNA levels and elevation of *Bax* mRNA in cervical and esophageal cancer cell lines were associated with apoptotic cell death induction of 5-aminolevulinic-mediated PDT [45,46]. PDT-mediated apoptosis in A431 cells resulted in significant deregulation of *Bcl-2* protein. The downregulation of *Bcl-2* sensitizes the PDT apoptosis-resistant cells to apoptosis [18]. A study by He et al., using a Chinese hamster ovary cell line, indicated that cells transfected with the *Bcl-2* gene were able to inhibit overall PDT death induced by silicon phthalocyanine. Their study found a high apoptosis incidence characterized by DNA fragmentation in parental cells, unlike in the transfected *Bcl-2* cells post- PDT [47]. Transfection of *Bcl-2* could lead to a higher *Bcl-2/Bax* ratio which inhibits initiation of apoptosis and consequently results in therapy resistance. Hence, these studies indicated the involvement of *Bcl-2* survival proteins in resistance response to PDT. 

## 5. Hypoxia-Induced Resistance to Photodynamic Therapy 

**Figure 2:**
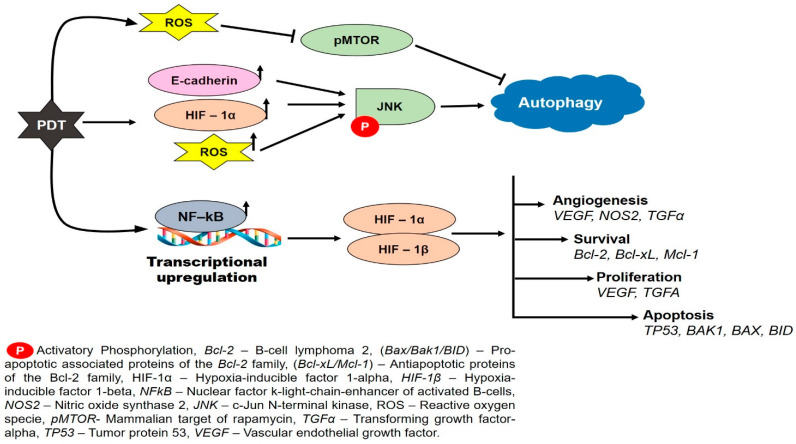
PDT-induced autophagy and resistance in hypoxic tumor. PDT is exacerbated by high levels of ROS, *HIF-1α*, and E-cadherin to activates c-Jun N-terminal kinase and cellular processes of autophagy. Alternatively, PDT in hypoxic tumor upregulates *NF-kB* to cause the stabilization of HIF-1 proteins and expression of angiogenic, surviving, proliferating signals that causes tumor relapse or apoptotic signal that kills the cell.

Hypoxia is one of the features in the tumor microenvironment that inhibit the therapeutic efficacies of chemotherapy, radiotherapy, and conventional photodynamic therapy (type II PDT) [48]. During cancer treatment, there is a high rate of oxygen consumption which influences the selection of malignant cells that are more aggressive, thus promoting the development of treatment-resistant cells [49]. Research has shown that hypoxic tumors are three times more resistant to therapeutic interventions such as chemotherapy, radiotherapy, and photodynamic therapy [50]. Tumor cells may protect themselves against PDT-mediated damage by stabilizing the hypoxia-inducible factor 1 (*HIF1*)-alpha (Figure 2). PDT induces hypoxia and vascular damage via the *HIF1*-alpha pathway, promoting tumor proliferation and survival [51]. PDT is exacerbated by high levels of ROS, which cause stabilization and activation of *HIF-1* proteins and expression of angiogenic, surviving, and proliferating signals (Figure 2) that result in tumor relapse [52]. To this effect, some innovations have been proposed to counter the negative influence of hypoxia during treatment. These approaches include using hyperbaric oxygen therapy, introducing external oxygen carriers such as perfluorocarbon and hemoglobin, in situ O_2_ generated catalysts such as photosynthetic bacteria and catalase, or O_2_ suppliers (CaO_2_). Other strategies to supply oxygen and inhibit glutathione (*GSH*) activity were adopted, such as catalytic reduction in hydrogen peroxide into intracellular oxygen by catalase enzymes or magnesium oxide to enhance PDT anticancer effects [53]. These approaches have made significant progress in developing better designs for effective PDT, thus showing promising usefulness of oxygen-independent cancer therapeutic approach [54]. 

Superoxide radicals are the main oxidant form of ROS that initiates from the mitochondria to stimulate DNA breakage and membrane injury through oxidative phosphorylation. They are converted to hydrogen peroxide by the enzymatic action of mitochondria superoxide dismutase (SOD) to trigger cellular apoptosis. Additionally, SOD could mediate O_2_ conversion further to a downstream toxic hydroxyl radical (OH), which aggravates treatment efficacy [55]. The tumor hypoxic treatment approach has aroused concerns for use to combat PDT resistance due to the oxygen consumption of PDT. Suppose an oxygen enrichment approach is used to supply O_2_ to hypoxic tumors during PDT. In that case, there will be an increased conversion of the O_2_ to transient free radicals, which deteriorates the tumor’s microenvironment and leads to strong cytotoxicity [56]. This has led to the recent development of PDT and the hypoxia-activated chemotherapy combination therapy approach reported by Wang and colleagues. This group investigated the approach by using an integrated nanocarrier system with 6-amino flavone (AF), an inhibitor of hypoxia-inducible factor-1α, to improve PDT resistance therapy in hypoxic tumors. They observed that the combination treatment activates the HIF-1 inhibitor AF to enhance the antitumor therapeutic effect [56].

## 6. Molecular Mechanisms of Cancer Resistance to Photodynamic Therapy

**Figure 3:**
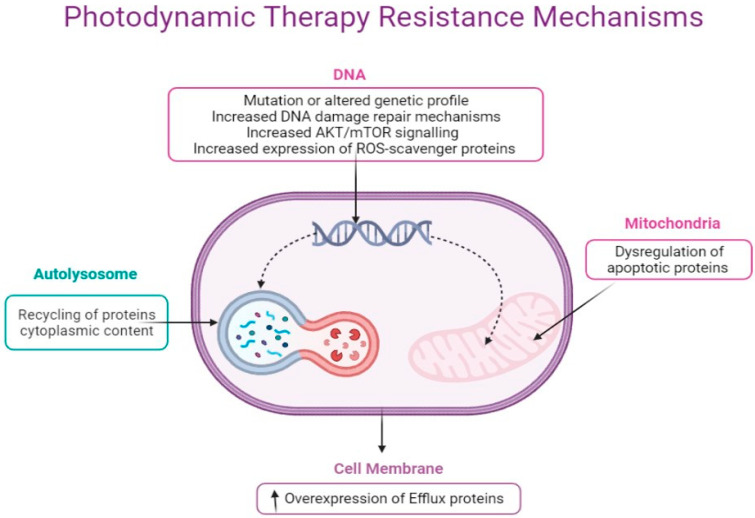
Cancer cells develop resistance to Photodynamic therapy through altered genetic profiling, increase in DNA damage repair mechanism, increase in *AKT/mTOR* signaling and expression of ROS-scavenger proteins. In the mitochondria, PDT can lead to dysregulation of antiapoptotic Bcl-2 protein at the cell membrane, overexpression of efflux proteins such as the P-glycoproteins, and autolysosome formation through recycling of cytoplasmic content for cell survival.

Cancer resistance to therapy is one of the major challenges in cancer and contributes to tumor metastasis. The PDT approach of using light energy, PS molecule, and molecular oxygen to destroy tumor cells has shown some levels of dormancy which, thus, have led to cell survival and phototherapeutic resistance. In essence, PDT, similar to other cancer therapies, can also elicit changes in the tumor microenvironment during treatment, leading to cancer cell adaptation and ultimately cell resistance [57,58,59]. Though the knowledge of tumor resistance to PDT is still at the infancy stage and have not really been documented, emerging studies have shown that poor delivery of PS and its photoexcitation, including the number of phototherapeutic sessions plus cancer cell type, are major determinants of tumor resistance to PDT [33,60]. Here, we briefly consider some of the main molecular mechanisms that underlie tumor defense against photooxidative damage and PS uptake, as summarized in Figure 3. 

Cancer cells develop intrinsic resistance to treatment due to some clonal selection changes prior to treatment [59]. These changes constitute an adaptive response to therapy-mediated stresses such as mutations and altered genetic and epigenetic profiles. Additionally, cancers can also develop resistance during treatment due to dysfunctional apoptosis, surrogate drug targets, increased drug efflux capacity, and activation of compensatory signaling pathways [14,61,62]. In this case, cancer cells are said to have acquired resistance, which affects the cytoskeleton and morphological appearance of the cell. This observation is linked to the impairment of migratory and invasive behaviors of cells that caused high cellular plasticity and cytoskeleton alteration [61]. The study of Lamberti and colleagues have shown oxygen depreciation and HIF-1α as major drivers of resistance in colorectal cancer cells during PDT. It was observed that these factors prevent the photosensitization of Protoporphyrin IX (PpIX) in a heterotypic spheroid cell model of colorectal SW480 cancer cells and fibroblast. This could not allow the endogenous production of PpIX but rather activate the *MAPK1/ERK2* and *MAPK3/ERK1* pathway as a compensatory adaptive survival during PpIX-PDT [62]. This adaptive pathway is due to the changes in the tumor’s microenvironment during treatment and, thus, acquired resistance. 

Cancer gene expression profile studies have identified drug-efflux proteins such as P-gp, breast cancer resistance, and multidrug-resistance proteins as mediators of most chemotherapeutic drugs [63,64,65,66]. These proteins belong to the ATP binding cassette (ABC) superfamily transporters whose function is to extrude foreign compounds such as PS and drugs out of the cells [67,68]. Based on these, mediation of PDT effectiveness in cancer therapy resistance could lead to the use of a nanotechnology delivery system approach to mitigate the efflux action of the resistance proteins on PS [63,69]. 

The antioxidant defense mechanisms and ROS-scavenger proteins such as glutathione S-transferase (GST) or glutathione peroxidase 4 (*GPx4*) have been shown as effectors of the phototoxic PDT effects on cells that leads to PDT resistance [70,71]. PDT utilizes ROS to effect damage to cells, and this defense machinery could also be activated during PDT therapy to quench ROS production to reduce the killing efficacy contributing to resistance. Likewise, the degree of heat shock proteins expression has been reported to be associated with an increase in autophagy [72] or apoptosis lessening [73], which ensure mediation of Photofrin-photooxidative resistance to tumor cells [74]. Additionally, nitric oxide (NO) generation through inducible nitric oxide synthase (*iNOS/NOS2*) during PDT has been reported to cause resistance. An interesting observation by Alderton and co-workers revealed that the photooxidative stress of PDT to some extent activates the induction of NO synthase that catalyzes L-arginine conversion to citrulline and NO. This process occurs in a Ca+2-independent manner and can prevent the killing efficacy of PDT [75]. 

Furthermore, is the role of *NFκB* via the increase in *AKT/mTOR* signaling activation in PDT, which has been observed in glioblastoma U87 and LN18 cells [76]. The 5-ALA-photoinduced stress stimulates tumor adaptation and resistance against 5-ALA-PDT photo-oxidation by activating the autophagic process through *AMPK* signaling pathway. Once this survival pathway is stimulated, a negative feedback regulation will decrease proapoptotic caspase-9 activity to suppress cell death [77]. In essence, tumor PDT resistance seems to correlate with ROS production which detects the activation of death or survival signaling pathways. Therefore, any process that alleviates proteotoxicity might lead to functional consequences of anticancer PDT immunity [78,79] as well as PDT sensitivity [80]. Hypericin-mediated PDT with low fluency initiates autophagy that restores cellular homeostasis and, thus, resistance to the phototoxicity of PDT [31]. Meanwhile, autophagy suppression due to *ATG5* knockdown increases tumor cell death in non-malignant cells such as fibroblasts. Thus, in the absence of autophagy, there will be a dysfunctional release of cytochrome c from the mitochondria which triggers apoptosis by caspase 3 and *PARP1* cleavage activation. 

All these mechanisms were delineated from several in vitro PDT experiments involving resistant cancer cell variants summarized in Table 1. Similar to the development of drug resistance with chemotherapy, PDT was used in a likewise manner through repeated cycles of treatment to generate a resistant sublime. Different photosensitizers, such as Photofrin, Pyropheophorbide-α methyl ester (MPPa), and 5-aminolevulinic acid (5-ALA) and its precursors, were used. The first study of PDT-resistant cell isolation was performed by Luna and Gomer from the mouse radiation-induced fibrosarcoma (RIF-1) cell line, following a repeated porphyrin (Photofrin II,)-mediated PDT treatments [81]. Continuous exposure of RIF-1 Fibrosarcoma cells to repeated PDT results in the generation of cells with high proliferative ability, otherwise known as PDT-resistant cells. These cells serve as a model for the study of PDT mechanisms of drug resistance. It is worth knowing that the process has thrown more light and expanded our understanding of the rate of PDT resistance [49]. 

The knowledge of the drug resistance mechanism is vague. Still, research has identified a few subcellular targets such as DNA, cell organelles such as mitochondria, lysosomes, Golgi apparatus, endoplasmic reticulum, enzymes, and their receptors as a direct effector of treatment. Modifying the sensitivity of these cellular PDT targets or their repair system would surely improve the current understanding of PDT resistance mechanisms. Meanwhile, different reports have indicated that resistance to PDT for the tumor cell lines is photosensitizer-specific [49]. The development of an in vitro resistant cell culture model allows more studies on minimally invasive methods such as PDT. It will help understand the molecular mechanism of cancer resistance, as exemplified by the studies shown in Table 1. 

## 7. Conclusions and Future Perspectives 

The development of a new photosensitizer and its incorporation in nanoparticles for PDT has led to a surge of interest in recent years. PDT triggers a photochemical reaction that produces a reactive oxygen species with a PS, laser light, and molecular oxygen. Cancer cells can develop resistance to chemotherapeutic agents, and this seems to be a continuous process that limits the efficacy of many drugs used in therapy. Natural products obtained from medicinal plant sources have a bright future in becoming an important alternative source of treatment for cancer drug resistance. This is due to its lower cost, toxicity, and natural abundance within our environment. Many reports have shown the anti-proliferative effect of plant extracts against breast, lungs, and colon cancer. At the same time, others have shown the important pharmacological applications of natural products in the treatment of several human diseases. Hence, it is necessary to consider and look towards using new molecules from natural sources in drug development with an unexploited mode of action to fight against cancer drug resistance. Another important factor is the molecular interaction between the heat shock proteins, such as the binding immunoglobin protein (*GRP78*), and the induction of molecular cytoprotective mechanisms in cancer cells. Protein constitutes roughly 2/3 of cell mass and is also a target for the action of ROS generated during PDT. Protein modification, such as fragmentation, multimerization, unfolding, or aggregation, has been reported in response to PDT.

Reports have recorded that the *GRP78* protein is being overexpressed in breast, prostate, liver, colon, and gastric cancers resistance to Adriamycin [86]. Hence, resistance to PDT can be mitigated by *GRP78* inhibitors such as genistein and salicylic acid that can suppress *GRP78* expression. Likewise, survivin, an inhibitor protein of apoptosis, its expression after PDT has been found upregulated to modulate apoptotic response by inhibiting the activities of caspases [87]. Survivin protein functions in complex signaling and cellular adaptations involved in cell survival. Ways should be developed to downregulate survivin expression, such as combining PDT with a survivin inhibitor to increase the apoptotic indexes and cytotoxicity of PDT. Nanoparticles could also be one of the solutions to mitigate PDT resistance [88]. Nanocarriers can be used to encapsulate PS to ensure its biodistribution and activation within the cellular targets. This will prevent sequestration of PS by the efflux proteins, a major limitation of PDT ROS generation and effectiveness. These strategies could be used to boost anticancer effects and mitigate PDT resistance.

## Figures and Tables

**Table 1:** Summary of in vitro Isolation of Cancer Cells Resistance to Photodynamic Therapy.

Photosensitizer	Methods Used in the Isolation of Resistant Cell Population	Cancer Cell Line Used	Features and Possible Mechanism of Resistance	References
Photofrin II	Short exposure (initial injury associated primarily with the plasma membrane) and long exposure to PII-PDT (associated with organelles and enzymes) damage.	RIF-1 Fibrosarcoma cells	Overlapping mechanisms of membrane-bound P-gp transport system amplification decreased DNA repair or altered biotransformation pathway.	[81]
Methyl-5-aminoleuvlinic acid (Me-ALA)	Red light doses and Me-ALA concentration was used after ten cycles of Me-ALA-mediated PDT. The survival criteria are PDT with a 5–15% rate.	Basal cell carcinoma	Resistance is dependent on the production of endogenous photosensitizer protoporphyrin IX and its cellular localization.	[82]
5-aminolevulinic acid (5-ALA)	PDT-resistant cell line was isolated following repetitive cycles of ALA-mediated PDT.	Glioblastoma (U-87 MG) cells	High repair efficiency of oxidative DNA damage, high activity of apurinic site endonuclease 1 (APE1), and increased expression of DNA damage protein kinase ataxia telangiectasia mutated (ATM).	[83]
Pyropheophorbide-α methyl ester (MPPa)	Repeated cycles of PDT with increasing doses of Pyropheophorbide-α methyl ester-mediated PDT.	Human osteosarcoma (MG63 and HOS) cell lines	High expression of CD133, antiapoptotic B-cell lymphoma (Bcl-xL and Bcl-2), multidrug resistance protein 1 (MRP1), and breast cancer resistance protein (ABCG2).	[84]
Methyl-5-aminolevulinic acid (Me-ALA).	Successive cycles of Me-ALA-mediated PDT. Treatment conditions that caused survival rate of 5–10% were used as selection criteria.	Squamous carcinoma cells	Increased expression of cell-substrate adhesion proteins (β1-integrin, vinculin) and phosphor-survivin.	[85]
Methyl-5-aminolevulinic acid (Me-ALA).	The irradiation dose that caused cellular death rate of 70–90% in parental cells was selected.	Human glioblastoma cells (T98 G).	High mRNA expression levels of Fibroblastic growth factor receptor (FGFR), epidermal growth factor receptor (EGFR), and β-platelet-derived growth factor receptor (*βPDGFR*).	[7]

## Data Availability

Not applicable.
